# Pharmaceutical health care and Inuit language communications in Nunavut, Canada

**DOI:** 10.3402/ijch.v72i0.21409

**Published:** 2013-08-05

**Authors:** Sandra J. Romain

**Affiliations:** 1 Department of Anthropology, University of Toronto, Toronto, Ontario, Canada; 2 Department of Anthropology, University of Toronto Scarborough, Toronto, Ontario, Canada

**Keywords:** drugs, prescription, medication, remote, delivery, culture, counselling, communication, language

## Abstract

**Background:**

Pharmaceutical communication is an essential component of pharmaceutical health care, optimally ensuring patients understand the proper administration and side effects of their medications. Communication can often be complicated by language and culture, but with pharmaceuticals, misunderstandings can prove particularly harmful. In Nunavut, to ensure the preservation and revitalization of Inuit languages, the *Inuit Language Protection Act* and *Official Languages Act* were passed requiring that all public and private sector essential services offer verbal and written communication in Inuit languages (Inuktitut and Inuinnaqtun) by 2012.

**Methods:**

While the legislation mandates compliance, policy implementation for pharmaceutical services is problematic. Not a single pharmacist in Nunavut is fluent in either of the Inuit languages. Pharmacists have indicated challenges in formally translating written documentation into Inuit languages based on concerns for patient safety. These challenges of negotiating the joint requirements of language legislation and patient safety have resulted in pharmacies using verbal on-site translation as a tenuous solution regardless of its many limitations.

**Results:**

The complex issues of pharmaceutical health care and communication among the Inuit of Nunavut are best examined through multimethod research to encompass a wide range of perspectives. This methodology combines the richness of ethnographic data, the targeted depth of interviews with key informants and the breadth of cross-Canada policy and financial analyses.

**Conclusions:**

The analysis of this information would provide valuable insights into the current relationships between health care providers, pharmacists and Inuit patients and suggest future directions for policy that will improve the efficacy of pharmaceuticals and health care spending for the Inuit in Canada.

Universal health care is a keystone of Canadian national identity. The Canada Health Act ensures that the majority of health services are publicly funded for all Canadians, with administration occurring at the provincial and territorial level. For aboriginal Canadians, health funding for some goods and services (e.g. vision care, dental care and pharmaceuticals) may also be covered by Health Canada's Non-Insured Health Benefits (NIHB) program (1). Despite this universality of health care, significant differences occur in the health status of aboriginal and non-aboriginal Canadians. First Nations populations experience higher rates of mental illness, suicide, diabetes, asthma, cardiovascular disease, tuberculosis, hepatitis, syphilis and HIV/AIDS than non-aboriginal populations (2). The interactions of complex geographical, cultural and historical factors influence these disparities and provide many challenges for the goal of attaining health equality for all Canadians.

The population of Nunavut is predominantly (85%) Inuit (3), with many still speaking the native languages and engaging in traditional lifestyles with time spent on the land and hunting. The celebrations of traditional knowledge and customs are culturally cohesive forces shown to minimize social problems within Aboriginal communities (4). As such, the government of Nunavut is highly committed to policy and programs that support Inuit culture and language. The *Inuit Language Protection Act* and *Official Languages Act* were passed in Nunavut to ensure the preservation and revitalization of Inuit languages. These legislative acts require that all public and private sector essential services (including pharmaceutical services) offer verbal and written communication in Inuit languages by 2012. Concern has been raised regarding challenges meeting the requirements of these legislative acts due to an inability to provide comprehensive health services in Inuit languages.

Health care in Nunavut is strongly influenced by the geographical challenge of remote fly-in communities and by the provision of care by non-Inuit health care providers. For many of the inhabitants of Nunavut, who refer to themselves as Nunavummiut, routine health care is provided by nurses in community health centres and nursing stations with limited access to physicians, specialists or pharmacists. This paper will examine the complex issues of one component of aboriginal health care, pharmaceutical communication among the Inuit of Nunavut, Canada. A multimethod research plan is outlined as the most appropriate and comprehensive way to investigate these issues and to inform future policy initiatives.

## Methods

### Role of pharmaceutical health care

Pharmaceuticals have become significant and essential components of health care. It is essential that pharmaceuticals are taken as intended to ensure that patients achieve the best health outcomes. Adverse drug events (ADEs) can result from the proper (e.g. unanticipated allergic reaction) or improper (e.g. incorrect dosage) administration of pharmaceuticals, but are significantly minimized through effective patient–provider communication. Notification of drug allergies or interactions and clear dosing instructions are two examples of how communication can reduce ADE risk. ADEs have significant negative implications on patients; identifying causes and solutions is of paramount importance to improving population health and maximizing the efficiency of health care.

ADEs are frequent, costly and often preventable occurrences. A US study calculated the hospital-based ADE rate to be 6.5 per 100 admissions (28% which were judged preventable), resulting in an excess $5857 per patient in hospital costs alone (5). Although data regarding the prevalence of outpatient ADEs are incomplete, a Canadian meta-analysis indicates that as many as 28% of all hospital emergency visits were drug related, with an estimated 70% being preventable and almost 30% due to patient noncompliance (6). In addition to the financial implications, ADEs result in increased patient morbidity and mortality through noncompliance, mistreatment and exacerbation of pre-existing medical conditions. Demographic shifts of aging populations and lifestyle factors are resulting in increasing chronic disease rates that are predominantly managed with pharmaceuticals. A greater volume of co-morbidities and associated prescriptions will undoubtedly result in a greater number of ADEs. Identifying opportunities to reduce these risks will therefore become increasingly crucial to health care in the future.

Health literacy contributes to an individual's ability to exercise ownership and control over their own health care and is defined as “the ability to understand (e.g. read, write and speak) health-related information” (7). Although closely associated with language proficiency, health literacy also incorporates elements of mathematical understanding to affect abilities such as interpreting nutrition labels and dosing formulae as well as administration instructions and healthy lifestyle advice. Low levels of health literacy contribute to increased health care utilization (e.g. hospitalizations) and lower levels of preventative care (e.g. immunizations) resulting in poorer health status and premature deaths (7). Health literacy is essential for reducing medication errors and ADEs. Low levels of health literacy can serve as a challenge to patients in every health care interaction, from accessing and speaking with health care providers, to understanding the labels on their prescription bottles.

Communication between patient and health care providers plays a key role in health care outcomes. Translating evidence-based medical knowledge into linguistically understandable and culturally meaningful patient information has been identified as an essential component of effective health care in Aboriginal communities (8–10). The most recognizable communication barrier in North America is patient–provider language differences, often when predominantly English-speaking health care providers are serving non-English speaking patients. Limited English proficiency (LEP) has been found to negatively affect patient health status through several pathways. LEP patients have been found to be less likely to have visited a physician or mental health professional in the previous year, and are less likely to have received a mammogram or influenza immunization (11). Hospitalized LEP patients are more likely to have adverse events and to suffer more significant physical harm as a result of these events than English proficient patients (12), and predictably, these events were found to be more likely the result of communication errors than for English proficient patients. LEP patients are often unable to effectively communicate about symptoms, side effects or catch errors, which might otherwise be prevented by English-speaking patients (13). Hospitalized patients, although a higher level of acuity, are less likely to be affected by communication barriers because of multidisciplinary staff and closer monitoring of response to therapy. This contrasts to the ambulatory patient who may rely solely on the pharmacist as the only opportunity for health information and to clarify and validate comprehension of medication instructions. Therefore, the opportunity and responsibility for understanding may be of greater impact in the pharmacist–patient interaction.

Health literacy related to pharmacy includes literature (brochures, promotional materials), labelling (affixed to bottles or drug information pamphlets) and counselling. Pharmacists fill prescriptions, but also ensure patients understand administration, dosage, side effects and contraindications. Compliance is not merely patients understanding how to take their medications when they leave the pharmacy; it is also important that they understand how their medications may affect them once taken. Understanding possible side effects has been found to increase compliance. LEP patients, who often report a lack of explanation of side effects (14), exhibit significantly higher levels of noncompliance (15). Patients must understand what consists of an unpleasant yet normal side effect versus one requiring medication adjustments or medical attention. This distinction can increase medication effectiveness through compliance and minimize the occurrence and/or severity of ADEs.

Labelling is one of the most significant pharmaceutical communication tools as this information includes the proper dosage, administration and warnings. Importantly, this is the only information available when patients are taking their medications away from health care providers. The vast majority of labelling is provided in English, where complex, multistep text is often misinterpreted by even English-speaking patients with lower literacy levels (16,17). LEP patients are twice as likely to self-report an ADE due to problems understanding instructions than English proficient patients (18). Some jurisdictions have sought to mitigate the challenge of serving LEP patients with translated pharmaceutical labels, often produced through commercially available computer software. Although translated labels are an improvement in service for LEP patients, studies have found inconsistent availability of translated labels in pharmacies (19,20), and alarmingly, significant levels of translation inaccuracy. Anecdotal evidence has identified cases of incorrect label translations resulting in serious patient illness and death (21,22). Some pharmacists have expressed reluctance to provide label translations as they are concerned for liability and patient safety if labels are not translated accurately and officially verified. A recent study has found significant improvements in patient understanding when translations are in simple terms (e.g. pill versus tablet), with clear time periods (i.e. morning, noon, evening, bedtime) and numeric characters are included (i.e. 2 versus two). This indicates that health literacy is more than the translation of words, but rather the acknowledgement and consideration of patient education levels and familiarity with health care information (23). Consistently available, simply worded and accurately translated pharmaceutical labelling would provide LEP patients with a valuable tool to ensure that they are properly self-administering their medications, allowing them to maximize health benefits and minimize ADEs.

Pharmaceutical counselling is an opportunity for pharmacists to examine patient therapy regimens to identify contraindications or interactions that might have been previously overlooked or might result from over-the-counter (OTC) medications. Patients are also able to confirm medication instructions and ask questions. These interactions benefit from clarity in language and understanding. When language barriers exist, pharmacists often resort to patient family members or staff to provide translation services. While patients have been found to prefer family members to translate due to their trustworthiness (24), the use of both family and/or staff as translators is problematic. Family members may heavily censor or filter information that is deemed unimportant, delicate, culturally inappropriate or discomforting, while staff may have limited pharmaceutical knowledge or training which compromises the accuracy of the translation (25). Non-professional medical translators have been observed to make significantly more errors of potential clinical consequence than professional translators. These errors most often involve omissions regarding drug allergies and dosing (26). Studies from several US cities show that while as many as 88% of retail pharmacies reported that they served non-English speaking patients daily who would benefit from translation services, only 36% were able to provide a non-English prescription label “most of the time” and only 32% were able to verbally communicate in another language “most of the time” (19,27). The ability to provide uninterrupted and professional translation services to LEP patients in pharmacies is proposed to enhance patient compliance and reduce ADEs.

Interactions between patients and pharmaceuticals are affected by both language and culture. Prescription pharmaceutical usage has been shown to vary with ethnicity and is affected by differences in beliefs and cultural backgrounds. An example of this is the generalized diminished confidence in the effectiveness of pharmaceuticals among Asian patients (28,29). Culture can affect attitudes and beliefs regarding gender roles and different types of illness. Attitudes of Chinese men towards mental illness result in significantly fewer prescriptions for antidepressants being filled than for “white” men. Attitudes of Chinese women on illness result in significantly fewer prescriptions as a whole than for “white” women (29). Patient motivations or desires to seek health care and access to health care differ among various ethnic groups. Medication use in children also differs with ethnicity even when a confounding factor such as access to ambulatory health can be controlled (30). Ethnicity can affect medication use in several ways: through requests for prescriptions, through beliefs regarding the efficacy of prescription or OTC pharmaceuticals, or through difficulty accessing pharmaceutical services.

Diet and nutrition for the Inuit of Nunavut is subject to some unique considerations related to pharmaceuticals. An Inuit traditional lifestyle (i.e. seasonal hunting, referred to as “country foods” among the Nunavummiut, with time spent on the land with family and/or other community members in “camps”) has been shown to affect food and nutrient intake (31). Country foods have been identified as having high nutritional values. However, not enough is known in regards to how this diet affects absorption of pharmaceuticals, or regulation of metabolic processes (e.g. blood sugar levels). Food insecurity (interrupted access to food) in the North is also an issue that is exacerbated by poverty and the high cost of store bought foods (referred to as “market foods” among the Nunavummiut) (32). The transition to a westernized diet is also correlating to an increase in chronic health effects (33,34). These factors, in combination with irregular timing of meals (a large meal due to successful hunting versus regularly scheduled meals) and accessibility to health care when on the land, are factors that may affect pharmaceutical need, compliance and effectiveness. Unscheduled meal times may decrease the effectiveness of some medications that are best absorbed with food, or may contribute to adverse events like nausea, bloating, abdominal pain and gastritis that can be minimized with food.

Aboriginal populations have experienced a history of cultural and linguistic persecution that has threatened and even annihilated numerous indigenous languages and is identified as an important source of many current and continuing social issues. While it is uncertain what exact combination of factors contributes to aboriginal health disparities in Canada, it is widely recognized that cultural discordance with Westernized health systems plays an important role.

## Results

### Role of language legislation

In 2008, the *Inuit Language Protection Act* and *Official Languages Act* were passed in Nunavut to ensure the preservation and revitalization of Inuit languages. These legislative acts require that all public and private sector essential services (including pharmaceutical services) offer verbal and written communication in Inuit languages by 2012. This policy directive is supported by research demonstrating that the development of language rights for the preservation of minority languages can have a positive effect on health and well-being, as well as positive economic impacts, not just for the minority, but for society as a whole (35). In Nunavut, there are two recognized Inuit languages. For communities in eastern Nunavut, service must be offered in Inuktitut, while in western Nunavut, service must be provided in Inuinnaqtun.

While the legislation mandates compliance, policy implementation for pharmaceutical services is problematic. In Nunavut, there are five retail pharmacies, yet not a single pharmacist or individual with any official pharmaceutical training in the territory is fluent in either of the Inuit languages. Currently, pharmacies rely on staff (without formal training in translation) to translate all verbal and written instructions to patients including dosage, side effects and contraindications. The negative implications of using non-professional translators have been previously outlined; however the use of Inuit translators, even without professional training does have distinctive benefits. Research has highlighted the unique cultural challenges of health communication with aboriginal patients where direct questions are frowned upon and 2-way communication is preferred, clearly a communication style which is not easily accommodated in westernized health care (36). Pharmaceutical inserts or monographs provided by the manufacturer are not available in Inuit languages. To understand this information, patients rely on translators to communicate what the translator believes is relevant about their medications. Due to this process, there is the potential for incomplete information. Retail pharmacists are unable to formally translate written documentation into Inuit languages without the ability to verify the authenticity of the translations. The challenges of negotiating the joint requirements of language legislation and patient safety have often resulted in pharmacies using verbal on-site translation as a tenuous solution regardless of its many limitations.

In Nunavut, remote, fly-in communities produce unique challenges in the provision of pharmaceutical services. Most communities do not have a pharmacist and many patients never have the opportunity to speak to a pharmacist. Health care is most often provided by nurses supported by visits from fly-in physicians and medical evacuation to larger health care centres (Iqaluit, Winnipeg or Ottawa) when required. Understaffing and lack of continuity of care (i.e. nursing staff rotations) have been identified as a challenge in remote Aboriginal communities. Specifically, continuity of care is necessary for the establishment of relationships beneficial to chronic care management and cultural awareness (10). Health centres in fly-in communities have access to a basic dispensary from which pharmaceuticals are available for urgent-care or temporary needs. Nurses are able to dispense these medications for immediate use; however, longer term and/or nonstandard pharmaceutical needs must be prescribed by physicians when on their rounds in the community. Prescribed medications are ordered from the closest retail pharmacy by health care staff (typically by fax) and are dispensed by the pharmacy and flown into the community to be picked up at the health centre by the patient. This multistep process provides many opportunities for the introduction of communication errors, even in English without the added layer of language translation. As the majority of health care providers do not speak Inuit languages, the use of translators or family members is heavily relied upon throughout patient care for Nunavummiut allophones (those whose native language is neither English nor French). As discussed earlier, at each level, linguistic and cultural filters bias and censor elements of information based on embedded concepts of relevance and cultural appropriateness.

While in some jurisdictions pharmaceutical counselling is mandatory, in Nunavut this is not the case. To improve access to pharmacists, a proposal is being considered to transfer pharmacy services for the territory to a telepharmacist service located in Ottawa. Although Telehealth services are used extensively in the North to address geographical challenges, research has highlighted the need to consider the benefits of linguistically and culturally concordant care, which is best provided through local cultural knowledge (37). Transferring pharmacy services outside the community (in this case outside the territory) may increase phone access to a pharmacist, but will not solve the problem of ensuring cultural sensitivity and dissolving the language barrier. Rural and northern communities are acknowledged as being underserved by pharmacists and although data for Nunavut are conspicuously and singularly absent among provinces and territories, research supports policies and initiatives that work to recruit and retain pharmacy staff within communities, to maximize service needs that can be met locally (38).

## Discussion

The complex issues of pharmaceutical health care and communication among the Inuit of Nunavut are best examined through multimethod research to encompass a wide range of perspectives (39). The analysis of various types and sources of information would provide valuable insights and suggest future directions for policy that will improve the efficacy of pharmaceuticals and health care spending for the Inuit in Canada.

First, an examination of the current situation in Nunavut in regards to pharmaceutical health care would provide a foundational understanding of policy and service delivery and how these are interacting with language legislation requirements. This information would include the following: national and territorial legislation and regulation; financial expenditures and responsibilities (i.e. who pays for pharmaceuticals); interviews with pharmacists and other key informants about operational practices; and an understanding of general trends in pharmacy health care in the territory. Shifting to an Ottawa-based delivery model intends to improve access to pharmaceutical resources but is likely to have cultural and financial consequences that require thorough evaluation prior to implementation. This comprehensive research would examine multiple factors currently affecting pharmaceutical health care delivery in Nunavut.

Second, a historical and jurisdictional comparative analysis considering the development of pharmaceutical policy in Nunavut and jurisdictional differences between Nunavut and other Aboriginal communities would provide a broad foundational understanding of current practices in the territory. Particular attention would be focussed on those communities serving Aboriginal populations in remote locations in Canada, and those specifically with Inuit populations. This information can be supplemented with research and policy analysis from other multilingual jurisdictions with circumpolar aboriginal populations – such as the Sami in Norway (of particular interest due to article 110a of the Norwegian Constitution charging the State responsible for the preservation of Sami language and culture) and Aleut/Na-Dené in Alaska. These comparisons may be useful to determine how to provide linguistically concordant care and to consider what support government and pharmaceutical companies may be able to provide. These analyses may serve to highlight positive initiatives working elsewhere and suggest transferable applications to Nunavut.

Third, an ethnographic study to observe *in situ* the interactions of health care providers and patients in remote fly-in communities in Nunavut will provide rich contextual data on the unique cultural and language challenges in these Inuit communities. The attitudes and beliefs of health care providers and their patients can be best understood through direct observation and discussion of these interactions. The quality and nature of pharmaceutical counselling patients may receive can also be directly observed and discussions with physicians serving the remote communities can reveal how ADEs are currently addressed and/or drug therapies are adjusted. Additionally, interviews with Inuit community members can provide information on the knowledge, attitudes and beliefs surrounding traditional lifestyle factors and how they may affect pharmaceutical effectiveness, compliance and/or ADEs. Ethnographic research would provide rich and comprehensive insight into the complex interactions between policy, administration and Inuit language and culture.

## Conclusions

A multimethod approach can provide a complete and thorough examination of pharmaceuticals in Nunavut from perspectives and intersections of legislation, policy, administration, finance, Inuit language and culture. By combining interviews, ethnography, policy and financial analyses, historical and cross-jurisdictional research, the findings would provide a comprehensive and rich source of information when considering future improvements to the provision of pharmaceutical care to Nunavummiut. Such research findings would have the potential to inform health policy in Nunavut and elsewhere and provide valuable insight for future health policy revision.

Pharmaceutical health care delivery is affected by the policies and administration of the communities served. In Nunavut, the unique culture and language of remote Inuit communities provides additional communication challenges, which may result in ADEs and noncompliance among patients. Multimethod research is an effective strategy to examine these complex issues and inform future policy initiatives to minimize these challenges in Nunavut.
